# Cardiac autonomic neuropathy associated with global metabolic risk status in individuals at risk of type 2 diabetes

**DOI:** 10.3389/fendo.2026.1794135

**Published:** 2026-04-10

**Authors:** Magdolna Z. Békeffy, Anna E. Körei, Adrienn Menyhart, Dóra M. Balogh, Karola Osgyán, Gergely Balaton, Klaudia Lipták, Péter Kempler, Viktor J. Horváth

**Affiliations:** 1 Department of Medicine and Oncology, Faculty of Medicine, Semmelweis University, Budapest, Hungary; 2 Department of Paediatric Dentistry and Orthodontics, Semmelweis University, Budapest, Hungary; 3 Department of Prosthodontics, Semmelweis University, Budapest, Hungary

**Keywords:** cardiac autonomic neuropathy (CAN), continuous glucose monitoring (CGM), heart rate variability (HRV), metabolic risk, prediabetes

## Abstract

**Introduction:**

In prediabetes and in individuals at increased risk of diabetes, data on the relationship between continuous glucose monitoring (CGM)-derived glycaemic burden and autonomic neural dysfunction remain limited. Therefore, the objective of the present study was to investigate whether early neuropathic changes in these populations are related to short-term glycaemic variability or broader metabolic risk.

**Materials and methods:**

Increased risk of prediabetes was defined by the Finnish Diabetes Risk Score (HbA1c<5.7% and FINDRISC ≥12 points: n=14; controls <12 points, n=12; and prediabetes by HbA1c 5.70–6.49% (n=15); total number of participants n=41). Associations between metabolic status, CGM-derived metrics and autonomic function (RMSSD, pNN50, E/I ratio) were assessed using univariable and multivariable models. Cardiac autonomic neuropathy (CAN) was analysed by logistic regression, metabolic status by multinomial regression.

**Results:**

CAN was present in 16 participants (39%). Age showed a borderline association with autonomic neuropathy (OR = 1.06 per year; p=0.059), which was attenuated after adjustment for metabolic status. Both increased risk of prediabetes and prediabetes groups were significantly associated with older age (RRR = 1.11 and 1.43 per year; p=0.03 and p=0.002, respectively). In regression models, increased prediabetes risk (OR ~8.4) and prediabetes (OR ~7.0) status emerged as potential determinants of CAN independent of age, although confidence intervals were wide. Among CGM-derived metrics, only mean interstitial glucose differed across metabolic groups, while no glycaemic marker was associated with CAN.

**Conclusion:**

In this exploratory pilot cohort, CAN appeared to be more closely associated with metabolic risk status than with short-term glycaemic variability. CGM-derived metrics did not predict autonomic or sensory neuropathy, suggesting that early neural impairment in prediabetes and increased diabetes risk may be more closely linked to broader metabolic risk factors beyond short-term glycaemic variability. Larger longitudinal studies are warranted.

## Introduction

Autonomic and peripheral sensory neuropathies, including cardiac autonomic neuropathy (CAN) and distal symmetric polyneuropathy (DSPN), are among the most frequent microvascular complications of diabetes mellitus and are associated with impaired quality of life, increased cardiovascular risk, and excess mortality (1–3). The prevalence of CAN in type 2 diabetes mellitus is generally estimated at approximately 15–20% in asymptomatic individuals, with substantially higher rates reported in those with longer disease duration or poor glycaemic control (4). Importantly, CAN may already be detectable in earlier metabolic stages: in individuals with prediabetes, reported prevalence ranges up to 11%, and in those with metabolic syndrome up to 24%, depending on diagnostic criteria (5, 6). These data suggest that autonomic impairment may emerge before the onset of overt diabetes, supporting the concept that neural impairment is not solely a consequence of chronic hyperglycaemia (7–9) but may be influenced by additional metabolic and anthropometric factors (10).

The widespread use of continuous glucose monitoring (CGM) introduced novel tools to characterise glycaemic burden beyond HbA1c, including indices of short-term glucose variability and time spent outside the target range. In patients with established diabetes, observational studies and meta-analyses reported associations between abnormal glycaemic variability and the presence of CAN and DSPN (11–14). However, findings have been inconsistent and causality remains uncertain, particularly given that established autonomic neuropathy itself may adversely affect glucose regulation through impaired hypoglycaemia awareness or altered gastrointestinal motility.

Importantly, most existing studies focusing on populations with manifest diabetes. In contrast, data on the relationship between CGM-derived glycaemic metrics and neuropathy in prediabetes or in individuals at increased diabetes risk but normal glucose metabolism are scarce. Therefore, it remains unclear whether early neural impairment in these populations is related to subtle alterations in glycaemic variability or is primarily driven by broader metabolic risk.

Accordingly, the aim of the present study was to investigate whether CGM-derived measures of glycaemic burden and variability are associated with the presence of CAN or DSPN in individuals with prediabetes and in those at increased risk of diabetes. We hypothesised that metabolic risk status, rather than short-term glycaemic variability, would be the principal determinant of early neural impairment in these populations.

## Materials and methods

### Study design and participants

This cross-sectional study was conducted between 1 March and 30 September 2025 at the Department of Internal Medicine and Oncology, Semmelweis University, Budapest, Hungary. Adults aged ≥18 years under outpatient follow-up were eligible for inclusion. The study was performed in accordance with the Declaration of Helsinki and applicable local regulations. All participants provided written informed consent prior to enrolment. Ethical approval was granted by the National Institute of Public Health and Pharmacology (NNGYK/07600-4/2025).

Exclusion criteria included any form of diabetes mellitus, known systemic autoimmune disease, chronic kidney disease (KDIGO stage III–V), untreated thyroid disease, New York Heart Association class II–IV heart failure, active infection or hospitalisation within 30 days prior to enrolment. Individuals with known neurological disorders affecting peripheral or autonomic function were not included.

### Clinical and laboratory assessment

Medical records were reviewed to evaluate cardiovascular comorbidities, including hypertension, hyperlipidaemia and major cardiovascular events. Smoking status was also recorded. Participants receiving beta-blockers or non-dihydropyridine calcium channel blockers were excluded due to their known effects on heart rate variability. Use of other antihypertensive agents was recorded. Most antihypertensive medications consisted of ACE inhibitors or ACE inhibitor-based combination therapy. Anthropometric measurements were obtained using calibrated instruments, including body weight, height and waist circumference. Blood pressure and heart rate were measured in seated position after 5-minute rest; the average of two measurements was recorded.

Participants completed the Hungarian version of Finnish Diabetes Risk Score (FINDRISC) questionnaire (15). [https://diabetes.hu/findrisk] Venous blood samples were collected after at least 12 hours fasting and caffeine intake, alcohol consumption and vigorous physical activity were also asked to avoid at least 12 hours prior the investigations. Serum glucose, total cholesterol, LDL cholesterol, HDL cholesterol, triglycerides levels, estimated glomerular filtration rate and HbA1c were analysed in the central laboratory of our institution.

### Assessment of autonomic function

Prior to HRV assessment, participants underwent the abovementioned procedure which last at least altogether 10–15 minutes. ECG recordings were completed after this period. As the study was conducted in a routine outpatient setting, strict standardisation of timing the measurements were not feasible. Cardiac autonomic function was assessed using ECG-based testing (CPNSS/DB device; MSB MET Kft., Balatonfüred, Hungary). Standard limb-lead electrodes were applied in the supine position. The protocol consisted of a 1-minute resting ECG recording, followed by continuous ECG recording during three cycles of deep inspiration and expiration (5 seconds each). ECG signals were acquired at a sampling frequency of 500 Hz and analysed without filtering.

All ECG recordings were visually inspected to verify correct R-peak detection and to exclude artefacts. RR intervals were exported and HRV parameters were calculated using predefined formulas in Excel. Short-term HRV analysis was performed using a 1-minute resting ECG recording. Time-domain parasympathetic indices including RMSSD (root mean square of successive differences) and pNN50 (percentage of successive NN intervals differing by more than 50 ms) were calculated (16, 17). The expiratory-to-inspiratory (E/I) ratio was derived from the deep breathing test. Frequency-domain and nonlinear HRV analyses were not performed. RMSSD and pNN50 were selected as established short-term indices of parasympathetic function (16, 18) SDNN, while reflecting overall variability, is less reliable in ultra-short recordings and was therefore not used as a diagnostic threshold (18–20). Calculated values were cross-checked against the device-specific software output; minor numerical differences were observed but did not affect diagnostic classification. Participants with clinically relevant rhythm or conduction abnormalities were excluded and referred for cardiological evaluation.

### Definition of cardiac autonomic neuropathy

Cardiac autonomic neuropathy (CAN) was defined as either (i) concomitant reduction in parasympathetic time-domain indices (RMSSD <45 ms and pNN50 <15%) during resting 1-minute recordings, or (ii) an expiratory-to-inspiratory (E/I) ratio <1.21 during deep breathing testing.

The selected HRV thresholds were determined based on published adult short-term HRV reference ranges and established clinical autonomic testing practices, taking into account the mean age of the study cohort. Given that HRV indices are influenced by recording duration, age, sex, and heart rate, and that absolute HRV values are not directly interchangeable across different recording lengths, these thresholds should be interpreted as pragmatic operational definitions within the context of ultra-short (1-minute) resting recordings rather than universally validated diagnostic cut-offs (16, 17, 21). Age-specific HRV thresholds were not applied.

### Assessment of sensory neuropathy

Distal symmetric polyneuropathy was evaluated using current perception threshold (CPT) testing with the same device. CPT was assessed bilaterally on the index finger and great toe at 2000 Hz and 5 Hz, reflecting large myelinated and small unmyelinated fibre functions, respectively. Predefined normal ranges were applied. The normal CPT range was 120–398 mA at 2000 Hz and 16–100 mA at 5 Hz; mean (± SD) values were 244.6 ± 23.4 mA at 2000 Hz and 53.0 ± 15.1 mA at 5 Hz (22, 23). Sensory neuropathy was defined by abnormal CPT values recorded bilaterally using at least one testing modality.

### Continuous glucose monitoring

After completion of the clinical and neurophysiological assessments, participants were fitted with a continuous glucose monitoring (CGM) system (CareSens Air; 77 Elektronika Kft., Hungary) on the posterior upper arm. Participants were instructed to maintain their usual lifestyle during the 15-day monitoring period.

CGM-derived metrics included mean interstitial glucose, (BG_average), coefficient of variation (CV), time in range (TIR), time above range (TAR), and time below range (TBR). Normoglycaemia was defined as interstitial glucose values between 4.0 and 10.0 mmol/L. CGM data were exported from the device-specific software platform in raw format and subsequently analysed using SPSS.

### Definition of metabolic status groups

Participants were stratified into three groups based on FINDRISC score and HbA1c values. Controls were defined as HbA1c <5.70% and FINDRISC <12. Individuals at increased prediabetes risk had HbA1c <5.70% and FINDRISC ≥12. Prediabetes was defined as HbA1c 5.70–6.49%, irrespective of FINDRISC score.

### Statistical analysis

Continuous variables are presented as mean ± standard deviation or median with interquartile range, as appropriate. Categorical variables are expressed as counts and percentages. Group comparisons were performed using one-way analysis of variance or Kruskal–Wallis tests, with appropriate *post hoc* analyses. Comparisons between participants with and without autonomic neuropathy were conducted using independent-samples t-tests or non-parametric equivalents.

Associations between age and metabolic status were analysed using multinomial logistic regression; control group served as the reference category. Predictors of autonomic neuropathy were explored using univariable logistic regression to minimise overfitting. Odds ratios (ORs) with 95% confidence intervals (CIs) are reported. HbA1c and mean interstitial glucose were not included simultaneously in regression models due to collinearity. Multicollinearity was assessed in multivariable models using variance inflation factors (VIF). No evidence of problematic multicollinearity was observed (all VIF values < 2). HRV parameters were analysed in their raw form; no logarithmic transformation was applied.

All statistical analyses were performed using SPSS version 27.0 (IBM Corp., Armonk, NY, USA). A two-sided p value <0.05 was considered statistically significant. Formal power calculation was not performed *a priori* due to the exploratory nature of the study.

## Results

**Figure 1:**
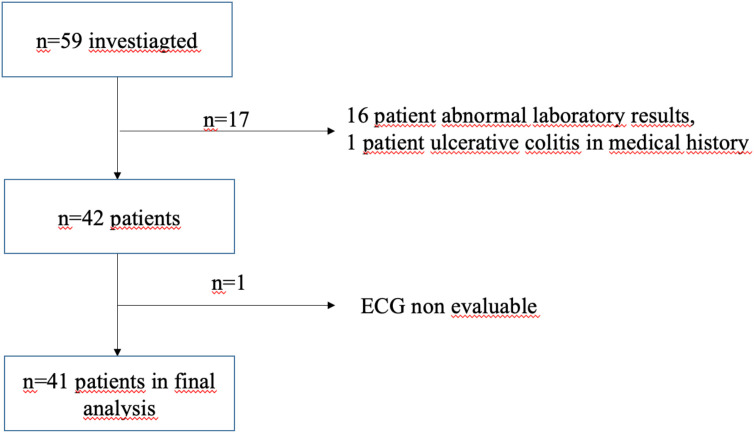
Flow diagram. Flow diagram illustrating participant selection and inclusion in the final analysis. Of the 59 screened individuals, 17 were excluded due to abnormal laboratory findings, elevated inflammatory markers, or relevant medical history. ECG-based autonomic testing was performed in 42 participants; one recording was excluded due to technical artefacts. The final analytical cohort consisted of 41 participants.

A total of 59 participants were initially screened (**Figure 1**). Exclusion criteria included elevated HbA1c values (>6.49%; n=10), abnormal thyroid-stimulating hormone levels (n=3), elevated inflammatory markers of unknown origin (n=3) and a documented history of ulcerative colitis (n=1). ECG-based autonomic testing was performed in 42 participants; one recording was excluded due to technical artefacts. The final analytical cohort therefore comprised n=41 individuals (n=20 men).

Of these, 12 participants (29%) were classified as controls, 14 (34%) as individuals at increased prediabetes risk and 15 (37%) as having prediabetes. Baseline clinical and laboratory characteristics stratified by metabolic status are summarised in **Table 1**.

**Table 1:** Baseline clinical and laboratory characteristics according to metabolic status.

Variables	Control (n=12)				Increased prediabetes risk + (n=14)				Prediabetes (n=15)				*p*
	Mean	SD	Upper CI	Lower CI	Mean	SD	Upper CI	Lower CI	Mean	SD	Upper CI	Lower CI	
Age (year)	34.2	7.5	30.0	38.4	43.8	11.0	38.1	49.6	57.5	9.0	52.9	62.0	**0.001**
Male sex, n (%)	5 (42%)				7 (50%)				8 (53%)				0.829
Waist (cm)	83.5	10.3	77.7	89.3	93.4	8.2	89.1	97.7	96.6	9.2	91.9	101.3	**0.003**
BMI (kg/m2)	23.3	2.8	21.8	24.9	26.4	3.1	24.7	28.0	30.0	6.1	26.9	33.0	**0.002**
SBP (mmHg)	123	10	117	129	133	16	124	141	136	9	131	140	**0.03**
DBP (mmHg)	73	8	69	77	78	10	73	83	82	8	78	86	**0,04**
Heart rate (beats/min)	62	12	55	69	69	11	63	75	65	7	62	69	0.23
TG (mmol/l)	0.8	0.3	0.6	1.0	1.5	1.5	0.7	2.3	1.9	1.7	1.0	2.8	0.13
Chol (mmol/l)	5.3	0.7	4.9	5.7	5.2	1.1	4.6	5.7	5.5	1.6	4.7	6.3	0.78
HDL (mmol/l)	2.0	0.4	1.8	2.2	1.5	0.3	1.4	1.7	1.5	0.5	1.2	1.7	**0.002**
LDL (mmol/l)	3.0	0.6	2.6	3.3	3.2	0.9	2.7	3.7	3.3	1.4	2.6	4.1	0.67
FBG (mmol/l)	4.8	0.3	4.6	4.9	4.9	0.4	4.7	5.0	5.3	0.8	4.9	5.7	**0.03**
HbA1c (%)	5.2	0.2	5.1	5.3	5.4	0.2	5.3	5.5	5.8	0.4	5.6	6.0	**0.001**
Sum of FINDRISC point	2	3	1	4	12	4	10	14	16	5	14	19	**0.001**
Hypertension, n (%)	0 (%)				4 (29%)				9 (60%)				**0.004**
Hyperlipidaemia, n (%)	1 (8%)				4 (29%)				10 (67%)				**0.006**
Current smoking, n (%)	1 (8%)				1 (7%)				0 (0%)				0.101
Mother DM, n (%)	1 (8%)				6 (43%)				4 (27%)				0.141
Father DM, n (%)	0 (0%)				3 (21%)				7 (47%)				**0.019**
Autonomic neuropathy, n (%)	1 (8%)				7 (50%)				8 (53%)				**0.034**
BG_average (mmol/l)	5.9	0.5	5.6	6.1	5.9	0.5	5.6	6.1	6.3	0.6	6.0	6.6	**0.04**
SD (mmol/l)	1.0	0.3	0.8	1.1	0.9	0.1	0.8	1.0	1.0	0.2	0.9	1.1	0.55
CV (%)	15.2	6.1	11.8	18.7	15.6	2.1	14.5	16.7	15.7	3.2	14.1	17.3	0.95
TIR (%)	98	2	97	99	91	24	79	104	91	23	80	103	0.61
TAR (%)	0	1	0	1	0	0	0	0	2	5	0	4	0.22
TBR (%)	2	2	1	3	2	2	1	3	1	1	0	1	0.17
RR average (msec)	1004	208	886	1122	856	277	711	1001	922	168	837	1007	0.25
RR SD (msec)	52	13	45	60	60	98	9	112	28	14	20	35	0.31
RMSSD (msec)	48	22	35	60	28	16	20	37	23	13	16	29	**0.002**
pNN50(%)	21	18	11	31	11	15	3	19	6	8	2	10	**0.032**
E/I	1.50	0.19	1.39	1.61	1.36	0.21	1.25	1.46	1.31	0.24	1.19	1.43	0.065
2KHz_RH (mA)	198	45	172	223	227	75	187	266	208	50	183	233	0.44
5Hz_RH (mA)	43	21	31	55	52	18	42	61	53	19	43	62	0.38
2KHz_LH (mA)	174	44	149	198	183	45	160	207	216	45	193	239	**0.045**
5Hz_LH (mA)	42	20	31	54	55	23	43	67	50	20	40	60	0.33
2KHz_RL (mA)	308	98	253	364	334	92	286	383	305	75	267	343	0.63
5Hz_RL (mA)	74	34	55	94	78	38	58	98	75	31	59	90	0.94
2KHz_LL (mA)	271	60	237	305	279	85	235	323	275	62	243	306	0.96
5Hz_LL (mA)	70	37	49	91	72	38	53	92	74	34	57	91	0.96

*BMI*, body mass index; *SBP*, systolic blood pressure; *DBP*, diastolic blood pressure; *Tg*, serum trigliceride; *Chol*, serum cholesterol; *HDL*, serum high density lipoprotein; *LDL*, serum low density lipoprotein; *FBG*, fasting blood glucose; *HbA1c*, serum glycated haemoglobin; *Sum of point*, summarized FINDRISC point; *DM*, diabetes mellitus (type 1 and 2); *BG_average*, average of CGM glucose levels; *SD*, standard deviation of BG_average; *CV*, coefficient of variance; *TIR*, time in range; *TAR*, time above range; *TBR*, time below range; *RR_average*, average distance of the consecutive RR distances in resting ECG; *RR SD*, standard deviation of RR distances in resting ECG; *RMSSD*, root mean square of successive differences; *pNN50*, percentage of consecutive heartbeats (NN intervals) that differ by more than 50 milliseconds; *E/I*, exspiration/inspiration ratio; *RH*, right hand; *LH*, left hand; *RL*, right leg; *LL*, left leg.

Baseline demographic, anthropometric, clinical, laboratory, continuous glucose monitoring-derived and autonomic function parameters of participants stratified by metabolic status (controls, increased prediabetes risk and prediabetes). Continuous variables are presented as mean ± standard deviation or median (interquartile range), as appropriate; categorical variables are shown as counts and percentages. Increased prediabetes risk group included only participants with FINDRISC ≥12; confidence intervals refer to the uncertainty of the group mean estimate. Bold values indicate statistically significant differences (p < 0.05).

Age, sex distribution, body mass index, waist circumference, systolic and diastolic blood pressure, and HDL cholesterol levels differed significantly across the three groups (all p<0.05). The prevalence of diagnosed hypertension, hyperlipidaemia and paternal history of diabetes was higher in both the increased prediabetes risk and prediabetes groups compared with controls.

As expected, based on group definitions, fasting plasma glucose and HbA1c differed significantly across groups. HbA1c showed a pronounced group effect (F = 17.62, p<0.001), with higher values observed in both metabolically abnormal groups relative to controls. Among CGM-derived metrics, only mean interstitial glucose differed significantly across metabolic groups (F = 3.54, p=0.039), driven primarily by higher values in the prediabetes group. No significant differences were observed for CGM-derived measures of glycaemic variability (CV, TIR, TAR, or TBR).

Regarding autonomic function parameters, RMSSD and pNN50 differed significantly across groups (both p<0.05), whereas the expiratory-to-inspiratory (E/I) ratio showed no significant between-group difference. The prevalence of CAN differed markedly across metabolic groups (controls: 1/9, 11%; increased prediabetes risk: 7/12, 58%; prediabetes: 8/15, 53%). Measures of sensory nerve function showed only minor, non-significant differences between groups.

### Characteristics according to autonomic neuropathy status

Based on predefined criteria, CAN was identified in 16 participants (39%) of the overall cohort (**Table 2**). Although mean E/I differed between groups, classification of CAN was driven by the predefined composite criterion and a substantial proportion of cases were identified by reduced RMSSD and pNN50 rather than an abnormal E/I ratio. Notably, individual-level classification was not based on mean E/I values; rather, reduced HRV indices (RMSSD/pNN50) accounted for a considerable proportion of autonomic neuropathy cases, explaining why group mean E/I may remain above the cut-off. When comparing participants with and without CAN, systolic blood pressure was significantly higher in those with neuropathy (p< 0.01) and hyperlipidaemia was significantly more frequent among participants with CAN compared to those without CAN (p=0.008) while other clinical, laboratory, and anthropometric variables did not differ significantly.

**Table 2:** Clinical, laboratory, and glycaemic characteristics according to autonomic neuropathy status.

Variables	Cardiac autonomic neuropathy								*p*
	Yes (n=16)				No (n=25)				
	Mean	SD	Upper CI	Lower CI	Mean	SD	Upper CI	Lower CI	
Age (year)	51.1	16.3	43.1	59.0	42.8	10.0	38.8	46.7	0.08
Male sex, n (%)	6 (38%)				14 (56%)				0.20
Waist (cm)	91.8	8.8	87.4	96.1	91.6	11.7	87.1	96.2	0.97
BMI (kg/m2)	27.4	4.5	25.2	29.7	26.4	5.4	24.3	28.5	0.51
SBP (mmHg)	138	13	131	145	126	10	122	130	**0.01**
DBP (mmHg)	80	9	75	84	76	9	73	80	0.25
Heart rate (beats/min)	69	12	63	75	63	9	60	67	0.10
TG (mmol/l)	1.6	1.6	0.8	2.4	1.3	1.3	0.8	1.8	0.53
Chol (mmol/l)	5.6	1.2	5.0	6.2	5.1	1.2	4.7	5.6	0.20
HDL (mmol/l)	1.6	0.6	1.3	1.8	1.7	0.4	1.5	1.8	0.50
LDL (mmol/l)	3.5	1.0	3.0	4.0	3.0	1.0	2.6	3.4	0.18
BG (mmol/l)	5.0	0.5	4.8	5.2	5.0	0.7	4.8	5.3	0.90
HbA1c (%)	5.6	0.3	5.5	5.8	5.4	0.4	5.3	5.6	0.05
Sum of point	13	6	10	16	9	7	6	12	0.08
Hypertension, n (%)	5 (31%)				8 (32%)				0.618
Hyperlipidaemia, n (%)	10 (63%)				5 (20%)				**0.008**
Current smoking, n (%)	1 (6%)				1 (4%)				0.453
Mother DM, n (%)	6 (38%)				5 (20%)				0.191
Father DM, n (%)	4 (25)				6 (24%)				0.612
Control, n (%)	1 (6%)				11 (44%)				0.034
Increased prediabetes risk, n (%)	7 (44%)				7 (28%)			
Prediabetes, n (%)	8 (50%)				7 (28%)			
BG_average (mmol/l)	6.0	0.6	5.7	6.3	6.0	0.5	5.8	6.2	0.86
SD (mmol/l)	1.0	0.2	0.9	1.1	0.9	0.2	0.9	1.0	0.61
CV (%)	16.2	2.9	14.8	17.6	15.1	4.5	13.4	16.9	0.35
TIR (%)	91	22	80	102	95	18	88	102	0.61
TAR (%)	2	5	0	4	0	1	0	1	0.30
TBR (%)	2	2	1	2	1	2	1	2	0.83
RR average (msec)	845	158	768	923	974	248	877	1071	**0.05**
RR SD (msec)	25	11	19	31	60	72	31	88	**0.03**
RMSSD (msec)	17	7	14	21	41	20	34	49	**0.001**
pNN50(%)	3	4	1	5	18	17	11	24	**0.001**
E/I	1.24	0.15	1.16	1.32	1.47	0.22	1.39	1.56	**0.001**
2KHz_RH (mA)	195	50	170	220	222	62	198	246	0.14
5Hz_RH (mA)	44	13	38	51	52	22	44	61	0.15
2KHz_LH (mA)	195	48	172	219	190	48	172	209	0.76
5Hz_LH (mA)	51	23	40	63	48	20	40	56	0.64
2KHz_RL (mA)	338	97	291	386	302	79	271	333	0.22
5Hz_RL (mA)	84	44	63	106	70	25	61	80	0.26
2KHz_LL (mA)	277	74	241	313	274	66	248	300	0.90
5Hz_LL (mA)	83	43	62	104	65	27	54	76	0.15

Comparison of demographic, anthropometric, laboratory, continuous glucose monitoring-derived, and cardiovascular parameters between participants with and without autonomic neuropathy. Continuous variables are presented as mean ± standard deviation or median (interquartile range), as appropriate. Abbreviations see at **Table 1**. Autonomic neuropathy was defined on an individual basis using a composite criterion (RMSSD/pNN50 and/or E/I threshold); thus, group mean E/I values may remain >1.21 even when participants meet HRV-based criteria. Bold values indicate statistically significant differences (p < 0.05).

Also, neither mean interstitial glucose (6.00 vs 6.03 mmol/L; p=0.865) nor HbA1c (5.40% vs 5.63%; p=0.052) differed significantly between participants with and without CAN, although HbA1c showed a strong trend toward higher values in the neuropathy group. No CGM-derived measures of glycaemic variability were associated with the altered autonomic functional status.

### Influence of age and metabolic status on autonomic neuropathy

In multinomial logistic regression analysis, age was significantly associated with metabolic status. Each additional year of age increased the relative risk of belonging to the increased prediabetes risk group (RRR = 1.11; 95% CI: 1.01–1.22; p=0.028) and the prediabetes group (RRR = 1.43; 95% CI: 1.15–1.79; p=0.002) compared with controls (**Table 3**).

**Table 3:** Association between age and metabolic status.

Status	RRR	Upper CI	Lower CI	P
*AGE (increased prediabetes risk vs. control)*	1,11	1,012	1,218	0,028
*AGE (prediabetes vs. control)*	1,432	1,146	1,79	0,002
AN Y/N	OR	Upper CI	Lower CI	P
AGE	1,055	0,998	1,115	0,059

Multinomial logistic regression analysis assessing the association between age and metabolic status, with the control group serving as the reference category. Results are presented as relative risk ratios (RRRs) with 95% confidence intervals. *AN Y/N*: presence (Y) or absence (N) of autonomic neuropathy.

In univariable logistic regression, age showed a borderline association with autonomic neuropathy (OR = 1.06 per year; p=0.059). However, after accounting for metabolic status, the association between age and autonomic neuropathy was attenuated and no longer significant (p=0.47). In these models, group membership emerged as the potential determinant of autonomic neuropathy: increased prediabetes risk status was associated with an approximately 8.4-fold increase in odds (p≥0.09) and prediabetes with an approximately 7.0-fold increase in odds (p=0.18) compared with controls, although confidence intervals were wide (**Table 4**).

**Table 4:** Predictors of autonomic neuropathy.

Status	OR	Upper CI	Lower CI	p
*Age*	1,02	0,95	1,11	0.542
*Increased prediabetes risk vs control*	8,89	0,81	97,68	0.074
*Prediabetes vs control*	7,43	0,44	124,76	0.164

Univariable logistic regression models evaluating the association between age, metabolic status, and the presence of autonomic neuropathy. Results are expressed as odds ratios (ORs) with 95% confidence intervals.

### Glycaemic burden, variability and autonomic neuropathy

In age-adjusted logistic regression models, neither mean interstitial glucose (OR = 0.73; 95% CI: 0.20–2.68; p=0.63) nor HbA1c (OR = 2.99; 95% CI: 0.36–25.12; p=0.31) was significantly associated with autonomic neuropathy (**Table 5**). Similarly, CGM-derived measures of glycaemic variability (SD, CV, TIR, TAR, and TBR) showed no significant associations in any model (all p=0.40). Model fit was acceptable (Hosmer–Lemeshow test: all p=0.14). Additional Spearman correlation analyses between CGM-derived parameters and HRV indices revealed no statistically significant associations (all p>0.05).

**Table 5:** Association between glycaemic markers and autonomic neuropathy.

Variable	OR	Upper CI	Lower CI	P
Age	1,06	0,998	1,127	0,058
Average BG	0,725	0,196	2,679	0,63
Age	1,038	0,974	1,105	0,248
HbA1c	2,986	0,355	25,115	0,314

Age-adjusted logistic regression analyses examining the relationship between CGM-derived glycaemic metrics, HbA1c, and the presence of autonomic neuropathy. Results are presented as odds ratios (ORs) with 95% confidence intervals.

The correlation between HbA1c and mean interstitial glucose was weak and non-significant (Spearman’s rho = 0.164; p=0.304), indicating that these markers captured partially distinct aspects of glycaemic exposure in this cohort.

### Sensory neuropathy measures

Quantitative sensory testing, including current perception threshold measurements at 5 Hz and 2000 Hz, revealed no statistically significant differences either across metabolic status groups or according to autonomic neuropathy status (all p>0.10). A modest trend toward higher thresholds was observed in the prediabetes group for selected parameters, but these differences did not reach statistical significance.

## Discussion

In this cross-sectional study, we investigated whether continuous glucose monitoring-derived measures of glycaemic burden and variability are associated with autonomic and sensory neuropathy in individuals with prediabetes and in those at increased risk of diabetes but preserved glucose metabolism. Given the limited sample size and the number of events, the present analyses should be regarded as exploratory and hypothesis-generating and effect size estimates should therefore be interpreted with caution. Despite these limitations, our findings indicate that metabolic risk status, defined by FINDRISC score and HbA1c category, showed a consistent association with CAN, whereas neither short-term glycaemic variability nor mean interstitial glucose levels were related to neural dysfunction in this population.

### Metabolic risk status as a determinant of autonomic neuropathy

A key observation of the present study is that metabolic risk status emerged as a relevant determinant of autonomic neuropathy, independent of age. Although the latter was associated with both metabolic status and CAN in univariable analyses, its effect was attenuated after accounting for group membership. As age constitutes a component of the FINDRISC score, partial overlap between metabolic classification and age cannot be excluded. Nevertheless, age-adjusted regression analyses indicated that group membership retained a stronger association with autonomic neuropathy than age alone, suggesting that the observed relationship cannot be explained solely by age. In contrast, both increased prediabetes risk status and prediabetes were associated with markedly higher odds of autonomic neuropathy compared with controls, albeit with wide confidence intervals due to the limited sample size.

These findings suggest that autonomic neuropathy in this population is not simply a function of ageing per se but may reflect the broader metabolic milieu accompanying increased diabetes risk and early dysglycaemia. This interpretation is consistent with previous reports demonstrating that autonomic and peripheral neural impairment may already be present in prediabetes and even in individuals with elevated diabetes risk but normal glucose tolerance (7–9). Our results extend these observations by indicating that such early neural changes may be more closely linked to overall metabolic risk than to conventional glycaemic markers alone.

### Glycaemic burden and variability in early neural dysfunction

Another important finding is the lack of association between CGM-derived measures of glycaemic variability and autonomic neuropathy. Although mean interstitial glucose differed across metabolic groups - being highest in individuals with prediabetes - neither mean glucose nor indices of variability (SD, CV, TIR, TAR, or TBR) discriminated between participants with and without CAN.

These results are in contrast with studies conducted in populations with established type 2 diabetes, where increased glycaemic variability and long-term HbA1c variability have been associated with both diabetic peripheral neuropathy and cardiovascular autonomic neuropathy (11–14, 24). However, most of those studies involved patients with longer diabetes duration, higher absolute glycaemic burden and a greater prevalence of advanced microvascular complications. In early stages of metabolic alterations, such as prediabetes or increased diabetes risk, the pathophysiological drivers of neural injury may differ.

Our findings suggest that in these earlier stages, neural dysfunction may reflect broader metabolic alterations not adequately captured by short-term CGM-derived metrics, including obesity, dyslipidaemia, low-grade inflammation, oxidative stress, and microvascular dysfunction (25, 26). These processes may precede overt dysglycaemia and may not be adequately captured by short-term CGM-derived metrics. Accordingly, short-term glycaemic variability does not appear to be the primary explanatory factor for CAN in our population.

It should also be noted, that CGM profiles differed only modestly across metabolic risk groups. Importantly, glycaemic variability in our cohort was modest, with high TIR values across all metabolic groups. This restricted range may have limited the ability to detect associations between CGM-derived metrics and neural dysfunction. Apart from higher mean interstitial glucose values in the prediabetes group, indices of glycaemic variability did not differ between controls, individuals with elevated FINDRISC scores, and those with prediabetes. This finding suggests that short-term CGM-derived glycaemic patterns may not adequately capture the metabolic alterations underlying early neural dysfunction, particularly in individuals classified as high risk based on clinical risk scores rather than glycaemic criteria.

### Autonomic versus sensory neuropathy

An additional observation is the dissociation between autonomic and sensory neuropathy measures. While CAN (based on the applied operational definition) was common – affecting nearly 40% of the cohort – quantitative sensory testing did not reveal significant abnormalities across metabolic groups or according to autonomic neuropathy status.

This pattern suggests that, in individuals with early metabolic derangement, neural impairment may predominantly manifest in the autonomic domain, whereas sensory abnormalities may either develop later or remain below the detection threshold of conventional testing methods at this stage. This interpretation aligns with previous literature indicating that CAN often precedes clinically detectable distal symmetric polyneuropathy, particularly in early diabetes and prediabetes (26, 27). The preferential vulnerability of autonomic fibres, especially parasympathetic cardiac innervation, may partly explain this temporal sequence. Because RMSSD and pNN50 are robust markers of parasympathetic modulation and are considered reliable in short-term HRV recordings, they were used as indices of cardiac autonomic function in the present study (18, 19). Our findings are also in line with the concept that early neural impairment in dysmetabolic states may reflect broader metabolic alterations not captured by short-term CGM-derived metrics. In a large cohort using standardized quantitative sensory testing, Tsilingiris et al. reported that early sensory deficits were independently associated with insulin resistance in individuals without diabetes and with metabolic syndrome and advanced glycation end-products in type 2 diabetes, and that these early deficits predicted subsequent peripheral neuropathy development (19). Together with these data, our results support a model in which early autonomic dysfunction in prediabetes and FINDRISC-defined increased type 2 diabetes risk reflects broader dysmetabolic burden rather than short-term CGM-derived variability.

Nevertheless, the absence of detectable sensory neuropathy should be interpreted with caution, as subtle sensory nerve damage may not be captured by current perception threshold testing in small samples (28, 29). Longitudinal studies incorporating more sensitive techniques may help clarify the temporal relationship between autonomic and sensory nerve involvement. Future studies should incorporate complementary methods for autonomic assessment beyond short-term HRV analysis, including standardised cardiovascular reflex tests (e.g., Valsalva manoeuvre, orthostatic blood pressure testing) or longer ECG recordings allowing frequency-domain analysis. Similarly, other, widely used tools for early sensory nerve dysfunction – such as vibration perception threshold testing, monofilament examination or quantitative sensory testing targeting small fibre function – may provide additional insight into subclinical neuropathy in metabolically at-risk populations.

### Clinical implications

From a clinical perspective, our findings suggest that individuals with prediabetes or elevated diabetes risk may already exhibit CAN before the onset of overt diabetes. Importantly, this dysfunction appears to be more closely related to overall metabolic risk than to short-term glycaemic variability. These observations support the potential value of early autonomic function assessment in high-risk populations, particularly among those with elevated FINDRISC scores.

Early identification of CAN may facilitate timely lifestyle and metabolic interventions, including weight reduction, optimisation of lipid profiles and blood pressure control, which could potentially mitigate progression of neural injury (4, 26, 30). CGM remains a valuable tool for glycaemic assessment – its role in predicting early neuropathy in non-diabetic populations appears limited based on the present data.

Importantly, our findings suggest that HRV-based assessment of autonomic function may provide clinically relevant information even in normoglycaemic individuals with elevated metabolic risk. As autonomic dysfunction may precede overt diabetes and occur independently of short-term glycaemic variability, HRV assessment could serve as an early functional marker of neural involvement in high-risk populations.

### Strengths and limitations

The strengths of this study include the detailed phenotyping of participants, the combined use of FINDRISC and laboratory-based criteria to define metabolic status and the simultaneous assessment of autonomic function, sensory nerve function, and CGM-derived glycaemic metrics. The use of CGM allowed for a comprehensive evaluation of short-term glycaemic exposure beyond HbA1c.

However, several limitations should also be acknowledged. First, methodological considerations regarding the definition of cardiac autonomic neuropathy based on ultra-short HRV recordings should be discussed. Although RMSSD and pNN50 are widely accepted short-term indices of parasympathetic activity, universally validated diagnostic cut-offs for 1-minute recordings are not available. HRV values vary according to recording duration, age, sex, and heart rate, and absolute values are not directly interchangeable across different recording lengths. The applied thresholds should therefore be interpreted as pragmatic operational definitions within the methodological context of the present study rather than universally validated diagnostic standards. The relatively small sample size limited statistical power and resulted in wide confidence intervals in regression analyses. Moreover, fixed HRV thresholds were applied without age-specific adjustment. Given the known age dependence of HRV parameters, residual confounding related to physiological age-related decline cannot be fully excluded. Overall, our findings should be regarded as hypothesis-generating. Although the observed associations consistently pointed in the same direction, the relatively small sample size limited the precision of effect size estimates. Given the limited number of CAN cases, logistic regression models may be subject to instability and wide confidence intervals. Although univariable modelling was chosen to minimise overfitting, the results should be interpreted cautiously. The cross-sectional design precludes causal inference and does not allow assessment of temporal relationships between metabolic changes, glycaemic variability and neural dysfunction. Additionally, CGM was performed over a limited time period and may not fully reflect longer-term glycaemic patterns relevant to nerve injury.

The potential influence of concomitant medications and comorbidities should also be considered. Although participants receiving beta-blockers or non-dihydropyridine calcium channel blockers were excluded due to their known direct effects on HRV, other cardiovascular therapies – particularly ACE inhibitor-based regimens – were present in a subset of participants. While these agents are not known to exert major direct effects on short-term HRV parameters, their use likely reflects greater underlying cardiometabolic burden. Residual confounding related to disease severity therefore cannot be fully excluded.

## Conclusions

In summary, our study demonstrates that in individuals with prediabetes and in those at increased risk of diabetes, CAN is common and appears to be more closely associated with global metabolic risk status than with short-term glycaemic variability. CGM-derived metrics did not predict the presence of autonomic or sensory neuropathy in this population. These findings underscore the importance of early metabolic risk assessment and suggest that CAN may represent an early marker of neural injury preceding overt diabetes. Larger, longitudinal studies are warranted to clarify the mechanisms underlying early neuropathy and to determine whether targeted metabolic interventions can prevent its progression.

Supported by Hungarian Diabetes Association.

## Data Availability

The datasets presented in this study can be found in online repositories. The names of the repository/repositories and accession number(s) can be found in the article/supplementary material.
